# An epigenetic regulator emerges as microtubule minus-end binding and stabilizing factor in mitosis

**DOI:** 10.1038/ncomms8889

**Published:** 2015-08-05

**Authors:** Sylvain Meunier, Maria Shvedunova, Nhuong Van Nguyen, Leonor Avila, Isabelle Vernos, Asifa Akhtar

**Affiliations:** 1 Cell and Developmental Biology Programme, Centre for Genomic Regulation (CRG), Dr Aiguader 88, 08003 Barcelona, Spain; 2 Universitat Pompeu Fabra (UPF), Dr Aiguader 88, 08003 Barcelona, Spain; 3 Max Planck Institute of Immunobiology and Epigenetics, Department of Chromatin Regulation, 79108 Freiburg im Breisgau, Germany; 4 Faculty of Biology, University of Freiburg, 79104 Freiburg im Breisgau, Germany; 5 Institució Catalana de Recerca i Estudis Avançats (ICREA), Passeig Lluís Companys 23, 08010 Barcelona, Spain

## Abstract

The evolutionary conserved NSL complex is a prominent epigenetic regulator controlling expression of thousands of genes. Here we uncover a novel function of the NSL complex members in mitosis. As the cell enters mitosis, KANSL1 and KANSL3 undergo a marked relocalisation from the chromatin to the mitotic spindle. By stabilizing microtubule minus ends in a RanGTP-dependent manner, they are essential for spindle assembly and chromosome segregation. Moreover, we identify KANSL3 as a microtubule minus-end-binding protein, revealing a new class of mitosis-specific microtubule minus-end regulators. By adopting distinct functions in interphase and mitosis, KANSL proteins provide a link to coordinate the tasks of faithful expression and inheritance of the genome during different phases of the cell cycle.

## Abstract (web summary)

The heptameric KAT8-associated nonspecific lethal complex consists of highly conserved chromatin modifier proteins. Here, the authors show a role for the members of the complex in regulating microtubule assembly during mitosis.

## Graphical abstract

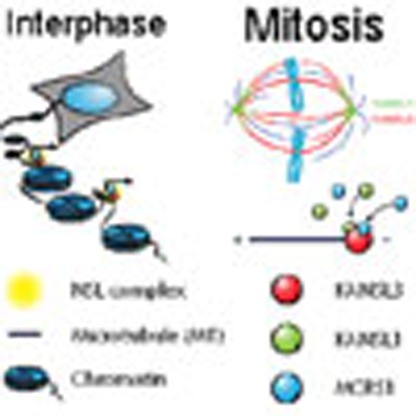

Chromatin modifiers are responsible for the establishment of ‘chromatin states' that determine the accessibility and activity of nuclear DNA. Different chromatin states are associated with altered organization and compaction of chromatin, and determine a given locus' transcriptional output.

The heptameric KAT8-associated nonspecific lethal (KANSL (mammals) or NSL (*Drosophila*); for simplification we will use ‘KANSL' throughout) complex is an important chromatin modifier in higher animals. Previous studies have determined that the KANSL proteins interact robustly both *in vitro* and *in vivo* and show high evolutionary conservation^1^,^2^,^3^. In *Drosophila* cells, the proteins are prominent regulators of housekeeping genes, with 89.4% of constitutively expressed genes bound by at least one KANSL protein^4^,^5^,^6^. In mammalian embryonic stem cells, these proteins additionally regulate enhancers and appear to be important for proliferation^7^,^8^.

KANSL proteins are essential in all the species in which they have been studied. *Drosophila* KANSL null mutants die latest in larval stages, and likely only survive thus far due to maternal contribution^6^. Misregulation of these proteins is additionally associated with diverse disease states in humans. A heterozygous mutation of the KANSL1 locus is sufficient to manifest in the Koolen-de Vries/KANSL1-related intellectual disability syndrome^9^,^10^,^11^ and mosaic single-nucleotide variations in KANSL2 were found to be frequently associated with severe intellectual disability in patients^12^. Thus far, the only known functions of the NSL complex have been described in interphase^5^,^7^.

During mitosis, a global rearrangement of chromatin structure takes place, leading to a totally unique chromatin state, the highly condensed ‘mitotic chromatin'. Although some chromatin modifiers remain associated with chromatin in mitosis, the vast majority of these factors are evicted, freeing them to perform functions in other cellular compartments^13^,^14^. In parallel, some nuclear proteins are known to play an essential role in the assembly of the mitotic spindle, by promoting microtubule nucleation and stabilization at the vicinity of the chromosomes^15^. This process is dependent on the small GTPase Ran, whose active GTP-bound form is concentrated around the mitotic chromatin^16^. A number of RanGTP-regulated spindle assembly factors have been identified so far^17^,^18^, including Imitation Switch (ISWI), which functions as a nucleosome remodeler in interphase^19^. It remains unclear how many other epigenetic complexes may have functions in mitosis not related to chromatin states or gene expression.

In this study, we describe the essential and novel contribution of KANSL1 and KANSL3 to spindle assembly. We found that KANSL1 and KANSL3 are novel microtubule-associated proteins that localize to the spindle poles during mitosis. Using *Xenopus* egg extracts to study their transcription-independent functions, we show that they interact with TPX2 in a RanGTP-dependent manner, promoting microtubule assembly *in vitro*. Moreover, we show that KANSL3 is an autonomous microtubule minus-end-binding protein. Our results suggest that KANSL3 targets other NSL complex members to K-fibre minus ends regulating their dynamics and performing an essential role in spindle assembly.

## Results

### KANSL1 and KANSL3 are required for cell division

**Figure 1:**
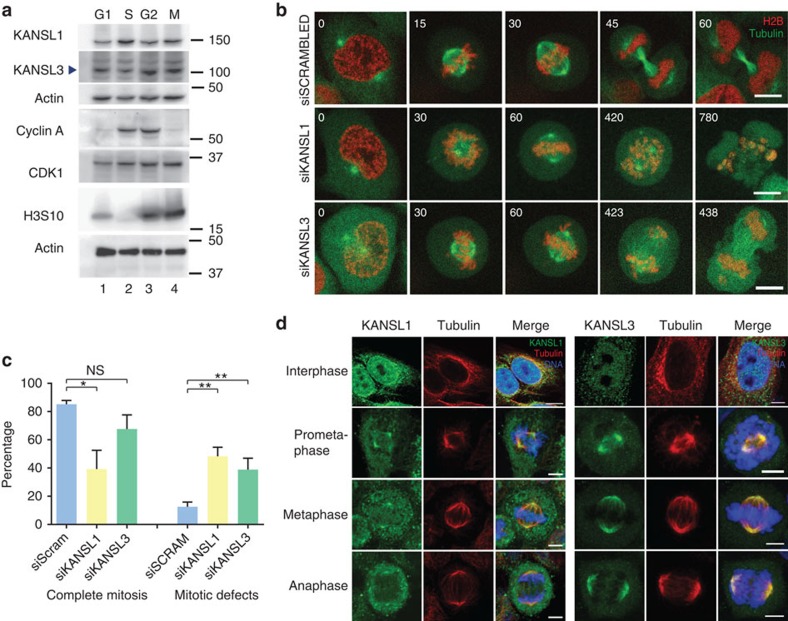
KANSL1 and KANSL3 localize to spindle poles in mitosis and their depletion leads to mitotic defects. (**a**) NSL complex members KANSL1 and KANSL3 are expressed throughout the cell cycle in HeLa cells. Synchronization was confirmed by western blotting of various cell cycle markers, as indicated. Molecular weight markers are indicated on the right and actin was used as a loading control. (**b**) Cells exhibit mitotic defects on knockdown of NSL complex members KANSL1 and KANSL3. Representative still images from the live-cell analysis show dividing control (siSCRAMBLED) and KANSL1- and KANSL3-silenced H2B-mCherry/a-tubulin-GFP HeLa cells. Typical phenotypes exhibited on knockdown of KANSL1 and KANSL3 include misaligned chromosomes persistently attached to spindle poles, a prolonged metaphase delay and mitotic catastrophe (note membrane blebbing in the final panel). Time in minutes is indicated in the upper left corners. Scale bars, 10 μm. (**c**) Left side: quantification of the percentage of cells that complete mitosis over a 24-h time frame. All other cells remained arrested in prometaphase or had died by the end of the observation period. Right side: quantification of the percentage of mitotic cells exhibiting the defects of misaligned chromosomes, multipolar spindles, lagging chromosomes or mitotic catastrophe. Error bars, s.e.m. *P<0.05, **P<0.01, NS, non significant, according to unpaired one-tailed *t*-test. (**d**) Immunofluorescence of KANSL1 or KANSL3 proteins (green) through the cell cycle. Tubulin is in red and DNA is in blue. Scale bars, 5 μm. KANSL1 and KANSL3 localize to spindle poles in mitosis.

To investigate whether the NSL complex could have additional roles in mitosis, we decided to focus on two of its core components, KANSL1 and KANSL3. To ascertain whether expression of NSL complex proteins is cell cycle regulated, we synchronized cells in discrete phases of the cell cycle with specific inhibitors and probed protein levels by western blot. We observed that KANSL1 and KANSL3 protein levels remained constant through all cell cycle stages (Fig. 1a; Supplementary Fig. 1a). Co-immunoprecipitation (co-IP) experiments in nocodazole-synchronized HeLa cells further showed that KANSL proteins were able to interact equally efficiently in mitosis as they do in interphase cells (Supplementary Fig. 1b).

Next, we investigated whether loss of NSL complex proteins had a consequence for mitotic progression. For this purpose, we used a cell line expressing mCherry-marked histone 2B and green fluorescent protein (GFP)-tagged tubulin^20^. We transfected cells with short interfering RNA (siRNAs; Supplementary Fig. 1c) and recorded stacks of images every 3 min over a 24-h period. Knockdown of KANSL1 or KANSL3 resulted in marked mitotic defects, the most prevalent of which was a prolonged arrest of cells in a prometaphase-like state (Fig. 1b; Supplementary Fig. 1d; Supplementary Movies 1–3). Following KANSL1 knockdown, 61% of cells entering mitosis were not able to complete it during the 24-h recording period (Fig. 1c). Cells silenced for KANSL3 had a less marked phenotype, possibly because it does not destabilize other members of the NSL complex such as KANSL1 silencing does (Fig. 1c; Supplementary Fig. 1c). A large proportion of KANSL1 and KANSL3 siRNA-treated cells exhibited misaligned chromosomes and various spindle organization defects (see Supplementary Movies and Supplementary Fig. 1e). The similarities of the phenotypes observed on knockdown of the two NSL complex members suggested a common function for these proteins during mitosis (Fig. 1b,c; Supplementary Fig. 1d,e; Supplementary Movies 4–6).

Having observed mitotic defects on their knockdown, we then used antibodies against KANSL1 and KANSL3 to investigate their subcellular localizations. In interphase, both the proteins were concentrated in the nucleus as expected (Fig. 1d, top panel). Interestingly, KANSL1 and KANSL3 exhibited a marked relocalisation during mitosis (Fig. 1d). In prometaphase and metaphase, KANSL1 was concentrated at spindle poles and in the pericentriolar region. The protein appeared then to remain associated with spindle poles throughout anaphase (Fig. 1d). Similarly to KANSL1, KANSL3 was strongly enriched at the spindle poles throughout mitosis (Fig. 1d). Staining specificity was verified by knockdowns (Supplementary Fig. 1f).

### KANSL1 and KANSL3 promote microtubule assembly *in vitro*

**Figure 2:**
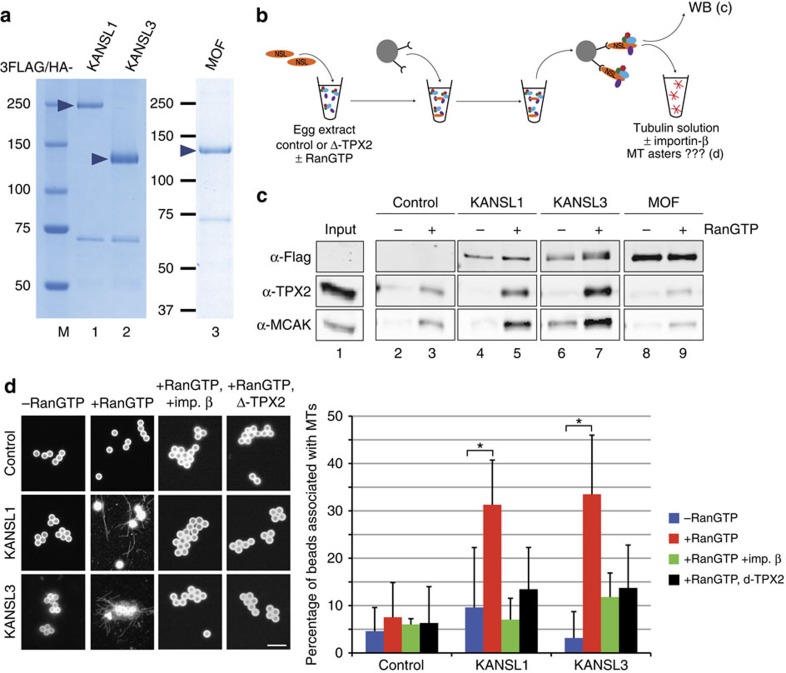
KANSL1 and KANSL3 promote microtubule assembly in a RanGTP-dependent manner in *Xenopus* egg extracts. (**a**) Coomassie blue-stained gel of purified recombinant *Drosophila* KANSL1, KANSL3 and MOF tagged with 3FLAG/HA. (**b**) Schematic representation of the experimental design. KANSL1 or KANSL3 was incubated in egg extract±RanGTP and retrieved on magnetic beads coupled with anti-HA antibodies. Beads were analysed by western blot or incubated in pure tubulin to monitor microtubule assembly. (**c**) Western blots of KANSL1, KANSL3 and MOF beads retrieved from M-phase *Xenopus* egg extracts in the presence (+) or absence (−) of RanGTP. The control was performed in parallel with egg extract in the absence of recombinant proteins. Anti-Flag antibodies were used to visualize KANSL1, KANSL3 and MOF. KANSL1 and KANSL3 interact with TPX2 and MCAK in a RanGTP-dependent manner. (**d**) Microtubule assembly activity in pure tubulin. The KANSL beads retrieved from M-phase *Xenopus* egg extracts in the presence (red bars) or absence (blue bars) of RanGTP were incubated in 20 μM pure tubulin. Green bars correspond to beads retrieved from an extract containing RanGTP and then incubated with importin-β and pure tubulin. Black bars represent beads retrieved from a xTPX2-depleted D-TPX2 egg extract containing RanGTP. Beads were spun down on coverslips and processed for immunofluorescence to visualize microtubules. Representative images of beads are shown on the left. Beads are autofluorescent. Scale bar, 5 μm. The graph on the right shows the percentage of beads interacting with microtubules in each condition. KANSL1- and KANSL3-coated beads induce the assembly of microtubules when retrieved from an egg extract containing RanGTP. Data are from three independent experiments. Error bars, s.d. *P<0.05 (unpaired *t*-test), NS, non significant.

The experiments above strongly suggested a role for KANSL1 and KANSL3 in mitosis. However, since the KANSL proteins regulate expression of housekeeping genes^4^,^5^,^7^, to separate this interphase function from a direct role in mitosis, we decided to take advantage of the *Xenopus laevis* egg extract system, in which transcription is totally inhibited^21^. Moreover, most of the core components of the NSL complex have been recently identified in a *Xenopus laevis* egg proteomic analysis^22^. We used three members of the *Drosophila* NSL complex that could be expressed and purified as full-length soluble proteins: 3FLAG/HA-tagged *Drosophila* KANSL1, KANSL3 and males absent on the first (MOF) (Fig. 2a). The proteins were individually incubated in egg extract either with or without exogenous RanGTP, and recovered by immunoprecipitation on magnetic beads. The beads were then washed and used for western blot analysis or placed in pure tubulin to test their microtubule stabilization or nucleation activities (Fig. 2b).

Interestingly, KANSL1 and KANSL3 beads, but not MOF beads, pulled down two important spindle assembly factors, TPX2 and MCAK, in a RanGTP-dependent manner (Fig. 2c). Moreover, the KANSL1 and KANSL3 beads retrieved from an extract containing RanGTP, but not from a control extract, promoted microtubule assembly in pure tubulin (Fig. 2d). In contrast, MOF-coated beads were unable to induce microtubule assembly in the same experimental conditions (Supplementary Fig. 2a). Since TPX2, a well-characterized mitotic protein, is known to be required for RanGTP-dependent microtubule nucleation^23^, we tested whether it was responsible for the microtubule assembly observed. We found that KANSL1 or KANSL3 beads retrieved from TPX2-depleted extracts containing RanGTP did not promote microtubule assembly *in vitro* (Fig. 2d). TPX2 is therefore essential for microtubule nucleation from KANSL1 and KANSL3 beads. However, incubation of KANSL1 or KANSL3 beads with an excess of importin-β abolished their microtubule assembly activity in this assay (Fig. 2d). Since importin-β does not bind directly to TPX2 and therefore does not inhibit its microtubule nucleation-promoting activity^24^, this indicates that other RanGTP-regulated protein(s) present on the beads additionally participate in the assembly of microtubules from KANSL1 and KANSL3 beads *in vitro.* We conclude that KANSL1 and KANSL3, in complex with spindle assembly factors, promote microtubule assembly in a RanGTP-dependent manner. This strongly suggests that both KANSL1 and KANSL3 have additional functions in mitosis related to microtubules and spindle assembly that are independent of their interphase role in transcription regulation.

### KANSL1 and KANSL3 regulate K-fibre minus-end dynamics

The RanGTP-dependent microtubule assembly activity of the KANSL proteins revealed by the egg extract experiments was reminiscent of the results described for an interphase KANSL-interacting protein, MCRS1 (ref. 25). However, MCRS1 also associates with other chromatin complexes in interphase^1^ and it is not known whether any of these interactions are maintained in mitosis. We therefore investigated this issue using a stable isotope labelling of amino acids in cell culture (SILAC)-based quantitative proteomic approach to identify MCRS1 mitotic partners. Five proteins from the NSL complex scored as specific partners of MCRS1 (Supplementary Fig. 2b). We confirmed these data by co-IP experiments from synchronized cells (Supplementary Fig. 2c). Consistently with our results in egg extract (above and ref. 25), we found that KANSL1, KANSL3 and MCRS1 also interact with TPX2 during mitosis in mammalian cells (Supplementary Fig. 2c).

**Figure 3:**
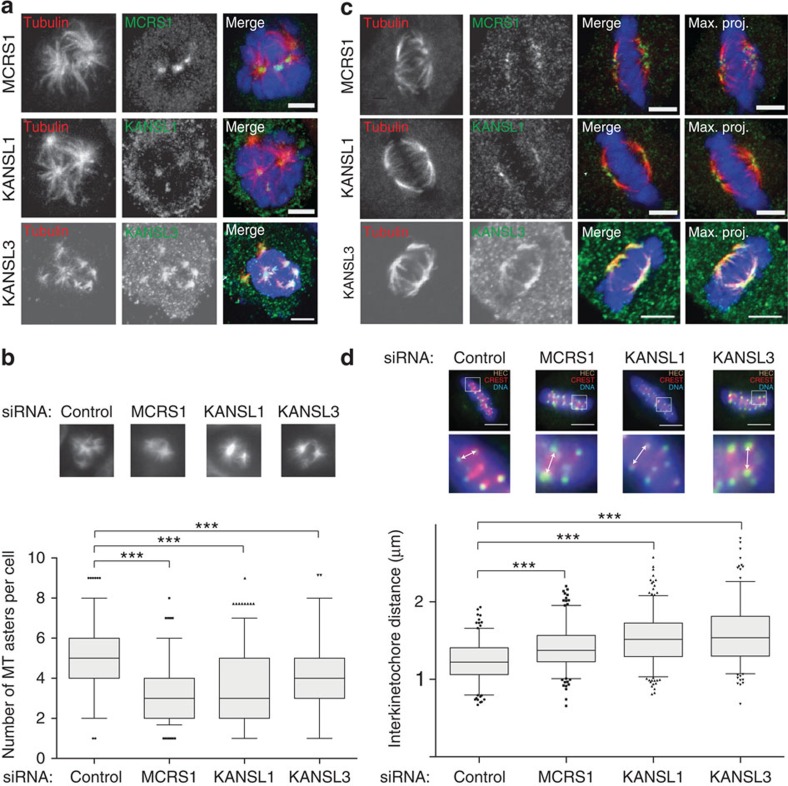
KANSL1 and KANSL3 are required for chromosomal microtubule assembly and K-fibre stabilization. (**a**) KANSL1 and KANSL3 localization to chromosomal microtubule asters. Immunofluorescence images of mitotic HeLa cells fixed 5 min after nocodazole washout. Tubulin is shown in red; MCRS1, KANSL1 and KANSL3 in green; and DNA in blue. KANSL1, KANSL3 and MCRS1 localize to the centre of microtubule asters. Scale bars, 5 μm. (**b**) KANSL1 and KANSL3 phenotypes in microtubule regrowth assay. Top: representative immunofluorescence images of microtubule asters in cells fixed 5 min after nocodazole washout. Bottom: number of microtubule asters. Box and whisker plot: boxes show the upper and lower quartile, whiskers extend from the 10th to the 90th percentile and dots correspond to outliers. *n*=3. MCRS1-, KANSL1- and KANSL3-silenced cells had on average a lower number of microtubule asters (3,429; 3,457 and 4,048, respectively), than control cells (4,996). More than 260 cells were analysed for each condition. ***P=0.0001 (unpaired *t*-test). (**c**) KANSL1 and KANSL3 localization to K-fibres. Cold-treated cells were fixed and stained with anti-tubulin (red) and anti-MCRS1, anti-KANSL1 or anti-KANSL3 (green) antibodies. DNA is in blue. Single confocal slices are shown except for the images on the right that correspond as indicated to maximum projections of 12 slices. KANSL1, KANSL3 and MCRS1 localize to the minus ends of K-fibres in mitosis. Scale bar, 5 μm. (**d**) Interkinetochore distance. Control, MCRS1-, KANSL1- and KANSL3-silenced HeLa cells were fixed and stained for the outer-kinetochore marker Hec1 (green) and CREST (red). The white arrows show paired sister kinetochores. The plot shows the interkinetochore distances in three independent experiments. Box and whisker plot: boxes show the upper and lower quartile, whiskers extend from the 10th to the 90th percentile and dots correspond to outliers. In MCRS1-, KANSL1- and KANSL3-silenced cells, the average interkinetochore distance is significantly increased (1,415; 1,504; 1,566 μm, respectively, versus 1,25 μm for the control cells). At least 221 kinetochores have been measured in each condition. Scale bar, 5 μm. ***P=0,0001 (unpaired *t*-test).

In view of the previously described function for MCRS1 (ref. 19), we decided to explore the function of KANSL1 and KANSL3 in the chromosomal pathway of microtubule assembly in human cells. For this purpose, we performed a microtubule regrowth assay, which can distinguish between the chromosomal and centrosomal pathways of microtubule nucleation in mitotic cells (see Methods)^26^. Immunofluorescence analysis on fixed cells showed that KANSL1 and KANSL3 localized to the centre of chromosomal microtubule asters similar to MCRS1 (ref. 25). In addition, KANSL1 and KANSL3 also localized to centrosomal asters (Fig. 3a; Supplementary Fig. 2d). We then examined whether KANSL1 and KANSL3 were involved in chromosome-driven microtubule assembly. Quantification of the number of microtubule asters in cells depleted of KANSL1, KANSL3 or MCRS1 during microtubule regrowth showed a statistically lower number than in control cells in line with our previous results on MCRS1 (on average 3 for MCRS1 or KANSL1 and 4 for KANSL3 knockdown, versus 5 in the control; Fig. 3b)^25^. These results strongly suggested that the KANSL proteins, in complex with MCRS1 play an important role in chromosomal-dependent microtubule assembly. Consistently, co-silencing of MCAK together with KANSL1 or KANSL3 led to a partial rescue in the number of asters in cells undergoing microtubule regrowth, again similarly to cells co-silenced for both MCAK and MCRS1 (Supplementary Fig. 2e)^25^. Taken together, these results suggest that KANSL1 and KANSL3 together with MCRS1 contribute to microtubule assembly in mitosis by stabilizing microtubules, likely by counteracting MT destabilizing activities.

One important property of MCRS1 is its specific localization to K-fibre microtubule minus ends^25^. We therefore examined the localizations of the KANSL proteins on K-fibres. HeLa cells were incubated on ice to induce the depolymerization of the more dynamic spindle microtubules, leaving behind only K-fibres^27^. Immunofluorescence analysis showed that KANSL1 and KANSL3 accumulated at K-fibre minus ends similarly to MCRS1 (Fig. 3c). This prompted us to look into the potential role of KANSL1 and KANSL3 in K-fibre dynamics. We first measured K-fibre length in KANSL1- and KANSL3-silenced cells and found that K-fibres were shorter than in control cells (Supplementary Fig. 2f). We then measured the interkinetochore distance in KANSL1- and KANSL3-silenced cells to test whether K-fibre dynamics was altered (Fig. 3d). We found that the distance was increased compared with controls. These data (Fig. 3d; Supplementary Fig. 2f) are consistent with what we described for MCRS1 (ref. 25) and reinforce the idea that KANSL1 and KANSL3 function in complex with MCRS1 to control the rate of K-fibre microtubule depolymerization at the minus end^28^. Taken together, we conclude that KANSL1 and KANSL3 interact and act together with MCRS1 in mitosis, stabilizing chromosomal microtubule and K-fibre minus ends.

### KANSL3 is a microtubule minus-end binding protein

**Figure 4:**
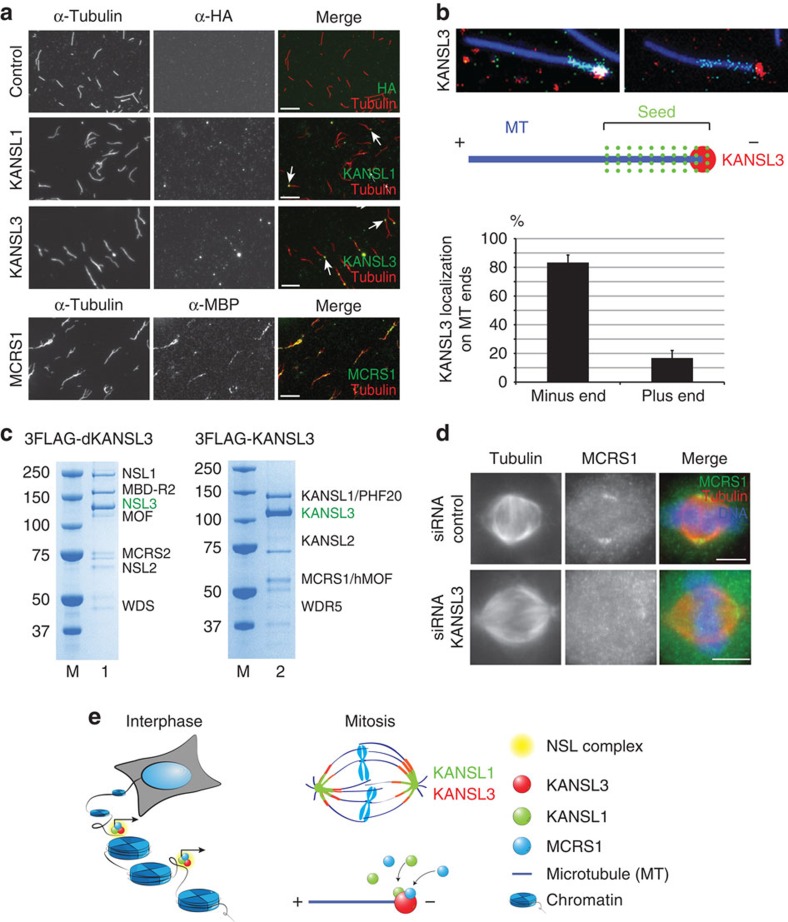
KANSL3 is a microtubule minus-end-binding protein. (**a**) KANSL1 and KANSL3 localization to microtubule ends *in vitro*. Taxol-stabilized microtubules were incubated with recombinant KANSL1, KANSL3, xMCRS1 or buffer (control), as indicated. Microtubules were spun down on coverslips and processed for immunofluorescence with anti-tubulin (red) and either anti-HA (for KANSL proteins) or anti-MBP (for MCRS1) antibodies (green). Arrows indicate KANSL1 and KANSL3 accumulation at microtubule ends. Scale bar, 5 μm. (**b**) KANSL3 binds to the minus end of polarity-marked microtubules *in vitro*. Polarity-marked microtubules were incubated with KANSL3, spun down on coverslips and processed for immunofluorescence. The bright microtubule seed at the minus end is in green, tubulin is in blue and KANSL3 in red, as shown in the drawing. KANSL3 associates with the microtubule minus end. Bottom: quantification of the microtubule minus end localization of KANSL3. The percentage of plus-end and minus-end signal for KANSL3 is shown. KANSL3 binds preferentially to minus ends (81.3%) versus plus ends (18.7%). *n*=3. More than 250 microtubules were analysed. Error bars, s.d. (**c**) KANSL3 nucleates a complete NSL complex *in vitro*. All seven members of the NSL complex were expressed in SF21 cells as untagged proteins except KANSL3 that was tagged with 3FLAG. Flag pull-down from the cellular extract retrieved a complete heptameric NSL complex. This indicates that KANSL3 is a structurally central component of the NSL complex, both in the context of the *Drosophila* and human proteins. (**d**) KANSL3 silencing in HeLa cells results in the loss of MCRS1 localization to spindle poles. Representative immunofluorescence pictures of control or KANSL3-silenced HeLa cells, as indicated. Tubulin is in red, MCRS1 in green and DNA in blue. Scale bar, 5 μm. (**e**) Summary. In interphase, KANSL1 and KANSL3 proteins are chromatin bound and regulate expression of housekeeping genes. During mitosis, these proteins relocate to the mitotic spindle and are important for cell division. Moreover, KANSL3 binds directly to microtubule minus ends *in vitro* and localizes to K-fibre microtubule minus ends in the dividing cell. Thus, KANSL proteins are able to adopt distinct tasks in different phases of cell cycle to ensure cellular homeostasis.

To better understand the interaction between the KANSL proteins and the mitotic microtubules, we first investigated their microtubule-binding properties *in vitro*. Recombinant KANSL1 and KANSL3 both co-pelleted with taxol-stabilized microtubules *in vitro*, while another member of the NSL complex, MOF, did not (Supplementary Fig. 3a). Since KANSL proteins localize to microtubule minus ends in mitosis (Fig. 1d and Fig. 3c), we then examined whether they associated preferentially with the ends of taxol-stabilized microtubules *in vitro*. As we showed previously, MCRS1 did not show any preferential end binding but associated all along the microtubule lattice (Fig. 4a). By contrast, 41% of microtubule-associated KANSL1 and 62% of microtubule-associated KANSL3 were indeed located at microtubule ends (Fig. 4a; Supplementary Fig. 3b–d). Moreover, they were preferentially associated with only one of the two microtubule ends (in 86% of the cases for KANSL1 and 81% for KANSL3, Supplementary Fig. 3e).

The specific end binding of KANSL proteins was particularly interesting, as very few proteins are known to bind directly the microtubule plus^29^ or minus end^30^,^31^,^32^,^33^,^34^,^35^,^36^. To determine which end of the microtubule KANSL3 preferentially associated with, we prepared taxol-stabilized, polarity-marked microtubules. In these microtubules, the minus end is clearly marked by an intense Rhodamine tubulin ‘seed'^37^. Strikingly, we found that KANSL3 preferentially associated with the microtubule minus ends in >81% of the cases (Fig. 4b). Taken together, these data indicate that KANSL3 is a novel direct microtubule minus-end-binding protein.

Since KANSL3 is capable of pulling down the entire NSL complex (*Drosophila* and mammalian) in *in vitro* reconstitution assays (Fig. 4c), we decided to test whether KANSL3 recruits MCRS1 to the microtubule minus ends in mitotic cells. In the absence of KANSL3, although MCRS1 levels are unaffected (Supplementary Fig. 1c), its localization to the spindle poles was indeed strongly reduced (Fig. 4d; Supplementary Fig. 4a). This was specific for MCRS1 as neither TPX2 nor NuMA localizations changed on KANSL3 silencing (Supplementary Fig. 4b,c). These data suggest that KANSL3 directly binds to the K-fibre microtubule minus ends and recruits MCRS1. Taken together, our results show that KANSL1, KANSL3 and MCRS1 constitute a mitosis-specific microtubule minus-end-associated complex essential for chromosomal microtubule assembly and correct K-fibre dynamics (Fig. 4e).

## Discussion

Earlier studies have considered the function of KANSL proteins exclusively in epigenetic regulation in the nucleus. Here we show that the NSL complex possesses a novel microtubule-binding and stabilizing activity in mitosis distinct from its interphase transcriptional activation activities. These functions are under the control of the RanGTP pathway, which allows the recycling of nuclear interphase complexes as microtubule regulators in mitosis. We describe a novel contribution of KANSL1 and KANSL3 to spindle assembly. Knockdown of KANSL proteins leads to marked and terminal mitotic defects, highlighting the essential nature of the KANSL proteins to cells. We show that KANSL1 and KANSL3 are able to regulate microtubule stability both in cells and *in vitro*. Our data strongly point to the existence of a functional microtubule-binding subcomplex consisting of at least KANSL1, KANSL3 and MCRS1 in mitotic cells. Although a few chromatin-binding factors have been shown to play a role in spindle assembly^18^, we show here for the first time that a chromatin-binding complex maintained in interphase and mitosis plays two distinct functions in these different cell cycle stages. Future work will determine whether the full NSL complex is involved in this mitotic function, or whether there are different specific subcomplexes. It is interesting to speculate that the cell may possess a large cytoplasmic pool of the NSL complex, likely stored in preparation for resumption of transcription in G1.

We also report the discovery of a new autonomous microtubule minus-end-binding protein, KANSL3. Thus far, only one protein family has recently been described to possess this property (namely Patronin/CAMSAPs)^30^,^32^,^36^,^38^,^39^,^40^. Since KANSL3 is predominantly nuclear in interphase, it may only associate with microtubules during mitosis. This makes KANSL3 a new microtubule minus-end-binding protein with a role in mitosis. Future work should help us to elucidate the structural elements that enable KANSL3 to recognize microtubule minus ends.

We propose that KANSL proteins play an important role in cellular homeostasis during different stages of the cell cycle. On the one side, they are transcriptional regulators targeting promoters of essential housekeeping genes during interphase and on the other side by stabilizing microtubules, KANSL proteins ensure faithful segregation of the genome during mitosis. Distinct contribution of these proteins during interphase and mitosis could also explain why cells as well as organisms (flies and humans) are sensitive to the dosage of KANSL proteins and their reduction or loss is not tolerated and is associated with microdeletion syndrome or cancer.

## Methods

### Cell culture

HeLa and HEK293 Flp-In Trex (Life Technologies) cells were grown in DMEM supplemented with Glutamax, 9% heat-inactivated fetal calf serum and 1 × penicillin and streptomycin (Life Technologies). The stable HeLa Kyoto cell line expressing GFP-tubulin and H2B-mCherry^20^ was maintained in the presence of 2 μg ml^−1^ puromycin (Carl Roth GmbH) and 500 μg ml^−1^ G418 (Sigma) until transfection. The stable HeLa cell line expressing GFP-centrin^41^ was maintained in the presence of 500 μg ml^−1^ G418 (Sigma).

A stable HEK293 cell line expressing FLAG-MCRS1 was generated by cotransfection of the pFlag-MCRS1 construct^25^ and the pBABE-puro vector (Addgene). Clones were selected using puromycin (2.5 μg ml^−1^; Sigma-Aldrich) and selected for FLAG-MCRS1 expression by western blot.

Stable inducible HEK293 Flp-In Trex (Life Technologies) cell lines carrying KANSL1-FBH (3XFLAG/biotin acceptor site/6XHis), KANSL3-FBH and MCRS1-FBH were generated by flippase recognition target (FRT) recombination according to the product manual. These cell lines were maintained in the presence of 15 μg ml^−1^ blasticidin and 150 μg ml^−1^ hygromycin. The day before synchronization, cell lines were induced by addition of 100 ng ml^−1^ doxycycline.

### Cell synchronization

For HeLa cell synchronization, cells were plated at 20% confluence and incubated with 3 mM thymidine (Sigma-Aldrich). After 16 h, cells were released into fresh medium for 8 h. Cells were then incubated with 3 mM thymidine for 14 h. For the ‘S-phase' sample, these cells were released into fresh medium for 2 h and then harvested by trypsinization. Part of the sample was reserved for fluorescence-activated cell sorting (FACS) and the other lysed in RIPA buffer (phosphate-buffered saline (PBS) with 1% NP-40, 0.5% sodium deoxycholate, 0.1% sodium dodecyl sulphate (SDS)), vortexed for 10 s and clarified by centrifugation at 10,000 r.p.m. for 15 min. For the ‘G2-phase' and ‘M-phase' samples, cells were released into fresh medium for 6 h and then incubated with 9 mM RO3306 (Alexis Biochemicals) or 50 ng ml^−1^ nocodazole (Calbiochem) for 8 h. G2-phase samples were harvested by trypsinization. M-phase samples were harvested by shake-off. For the G1 sample, cells were released into fresh medium for 8 h and subsequently incubated in 20 mM lovastatin (Santa Cruz) for 8 h. The cells were harvested by trypsinization.

HEK293 Flp-In Trex cells were synchronized in mitosis by incubation for 14 h with 10 μM (+)-S-trityl-L-cysteine (STLC, Alfa Aesar)^42^.

### FACS

Cells were washed in PBS once and then fixed in cold 75% ethanol with rotation overnight. Cells were stored at 4 °C until needed. For analysis, cells were stained with propidium iodide, treated with 0.2 mg ml^−1^ RNase A and observed on a BD LSRII instrument. Around 10,000 to 15,000 cells were measured per condition.

### Antibodies

Immunofluorescence and immunoprecipitations of KANSL1 and KANSL3 were performed using antibodies previously described^3^. Immunofluorescence for MCRS1 was performed using an antibody previously described^25^.

The following primary antibodies were used for immunofluorescence: α-tubulin DM1A (Sigma T9026; 1:1,000), α/β-tubulin (Abcam ab44928; 1:1,000), α-tubulin (Merck Millipore 04-1117; 1:400) and HA (Covance MMS-101P; 1:100). The MCAK polyclonal antibodies were a gift from E. Karsenti lab; the polyclonal Hec1 (9G3.2 GeneTex) antibody was used at 1:100 for immunofluorescence; CREST antibodies were used at 1:100 (Antibodies Incorporated 15-235-F); and the polyclonal MBP, hTPX2, NuMA and xTPX2 antibodies were produced in the Vernos lab.

The following primary antibodies were used for detection by western blot. All antibodies were used at a 1:1,000 dilution except where otherwise stated. KANSL1 (Abnova corporation PAB20355), KANSL3 (Sigma-Aldrich HPA035018), GAPDH (Bethyl Laboratories A300-641A, 1:6,000), Rad21 (Merck Millipore 05-908), Cyclin A (Abcam ab38), CDK1 (Abcam ab18), H3S10 (Abcam ab5176; 1:10,000), beta-actin (CST #4967; 1:3000), DM1A for α-tubulin (Sigma T9026; 1:10,000) and M2 for FLAG tag (Sigma F3165). The polyclonal hTPX2 antibody was produced in the Vernos lab.

Secondary antibodies (anti-mouse, anti-rat, anti-rabbit or anti-human) conjugated to Alexa488, 555, 568, 680 or 647 (Molecular Probes) were used at 1:500–1:1,000 for immunofluorescence and 1:10,000 for western blot.

### Co-immunoprecipitation in HeLa and HEK293 cells

Cells were harvested either by shake-off (for M-phase samples) or trypsinization (for unsynchronized samples). The contents of a 15-cm tissue culture plate were resuspended in 2 ml of high Tween20 HKMGT buffer (25 mM HEPES pH 7.6, 150 mM KCl, 12.5 mM MgCl_2_, 10% glycerol, 0.4% Tween20) with Complete protease inhibitors (Roche) added. The sample was sonicated for a total of 50 pulses output 3 duty cycle 30% on a Branson sonifier 250 with a microtip and subsequently clarified by spinning at 15,000*g* for 15 min at 4 °C. The cleared lysate was incubated either with immunoprecipitating antibody or directly with 30 μl pre-washed FLAG beads (Sigma) overnight. For endogenous co-IP, cleared lysates were incubated overnight with 5ul of antibody (see above) and then 1 h with pre-washed mixed ProtA and G beads. Endogenous co-IPs were washed three times in the lysis buffer. HEK FLAG IPs were washed twice in a low Tween, low stringency buffer (25 mM HEPES pH 7.6, 75 mM KCl, 12.5 mM MgCl_2_, 10% glycerol, 0.1% Tween20).

### Western blot analysis

Western blots in Fig. 1 and Supplementary Figs 1 and 2 were performed as follows. Following transfer, the 0.45 μm polyvinylidene difluoride membrane (Roche) was blocked for 1 h at room temperature in 5% bovine serum albumin (BSA) in PBS with 0.05% Tween20 (Sigma). Primary antibodies were incubated in 2% BSA, 0.05% Tween20 in PBS at 4 °C overnight. Secondary antibodies conjugated with horseradish peroxidase (GE Healthcare) were incubated in the same buffer at room temperature for 1 h.

Western blots in Fig. 2 and Supplementary Fig. 3 were performed using 0.45 μm nitrocellulose membrane (Whatman). Blocking and antibody incubations were performed using PBS with 3% milk and 0.1% Tween20 (Serva). Primary and secondary antibodies were incubated 1 h at room temperature. Secondary antibodies conjugated with AlexaFluors (see above) were used.

Uncropped western blots are presented in Supplementary Fig. 5.

### RNA interference

Cells were seeded at 15,000 cells per six well the day before transfection. Three-hundred picomoles of siRNA and 15 μl of XtremeGene siRNA transfection reagent (Roche) were used per six well (and scaled appropriately for smaller volumes). Transfection was performed as per supplier protocol. siRNA duplexes were ordered from MWG Operon using the following sequences (a mix of sequences 1+2 was prepared for transfection):

KANSL1 (1): 5′-CGGCAACGCCAACAUCCUU-3′

KANSL1 (2): 5′-GAAGCGGAGGCUUGUUCGA-3′

KANSL3 (1): 5′-GGCACGCAGCGTGATGAAT-3′

KANSL3 (2): 5′-GGAUGCUUGUGUCAUCCAA-3′

MCRS1: 5′-GGCAUGAGCUCUCCGGAC-3′

MCAK: 5′-GAUCCAACGCAGUAAUGGU-3′

MCRS1 and MCAK siRNAs were previously validated^25^.

### Immunofluorescence

For KANSL1 and KANSL3 immunofluorescence in Fig. 1d, Supplementary Figs 1f and 3d and KANSL3 immunofluorescence in Fig. 3, cells were grown on precision no. 1.5 coverslips (Carl Roth LH24.1) in six-well plates. Cells were pre-lysed for 20 s in PHEM (60 mM PIPES, 25 mM HEPES, 10 mM EGTA and 2 mM MgCl_2_) buffer with 0.5% Triton X100, washed once in PHEM buffer and fixed in 3.7% formaldehyde (Sigma F8775) in PHEM buffer with 0.1% Triton X100 at room temperature for 8 min. Cells were blocked in 2% BSA in PHEM +0.1% Triton X100 for 30 min at room temperature. Primary antibodies were incubated in blocking buffer overnight at 4 °C. Secondary antibodies conjugated with AlexaFluors (see ‘Antibodies' above) were diluted 1:500 in blocking buffer and incubated at room temperature for 1 h. Where appropriate, Hoechst 33342 (Life technologies) was added in the secondary antibody mix to stain DNA. Coverslips were mounted using Fluoromount G (Southern Biotech).

These samples were visualized with a 63 × objective on a Zeiss Observer.Z1 with the Yokogawa CSU-X1 spinning disk head and the Zeiss Axiocam MRm camera, with the exception of Fig. 1d KANSL3 immunofluorescence, which was visualized on the Zeiss LSM780 confocal microscope.

For KANSL1 immunofluorescence in Fig. 3 and all other immunofluorescences in Figs 3 and 4 and Supplementary Figs 2 and 4, cells were grown on coverslips in six-well plates and fixed in −20 °C methanol for 10 min. Blocking and antibody dilution buffer were 0.5% BSA (Sigma) and 0.1% Triton X100 (Sigma) in PBS. Primary and secondary antibodies were incubated for 1 h at room temperature. Coverslips were mounted in 10% Mowiol (Calbiochem) in 0.1 M Tris-HCl at pH 8.2, 25% glycerol (Merck).

For immunofluorescences in Figs 2 and 4a/b, samples were spun down at 1,000*g* for 10 min on coverslips before fixation in −20 °C methanol for 10 min.

These samples were visualized with a 63 × objective on an inverted DM1-6000 Leica widefield fluorescence microscope. Pictures were acquired with the Leica Application Suite software. Images were processed with ImageJ and Photoshop (Adobe) and assembled as figures using Illustrator (Adobe). Line scans for fluorescence intensity quantification along the spindles were performed using ImageJ.

### Live-cell imaging

Cells were seeded and treated in eight-well coverslip-bottomed dishes (ibidi GmbH cat. no 80826). Two days following transfection, dishes were transferred to a temperature- and CO_2_-controlled Tokai Hit stage incubation unit. Samples were visualized using a Zeiss Observer.Z1 with the CSU-X1 spinning disk head (Yokogawa) and the QuantEM:512SC camera (Photometrics). Z-stacks of cells were taken at 3-min intervals using a water immersion 63 × objective. For quantifications additional videos were taken at 8-min intervals using a 40 × long working distance objective. Time lapse was performed for a 24-h duration. Laser power and exposure times were kept to an absolute minimum.

### Recombinant proteins

Recombinant proteins were purified either from *Escherichia coli* or SF21 cells.

RanQ69L and x-importin-β were tagged with His-tag, produced in *E. coli* and purified using Ni-NTA agarose beads (Qiagen). RanQ69L was then loaded with 1 mM GTP in PBS 2 h at 4 °C. MBP-tagged x-MCRS1 was produced in *E. coli* and purified^25^ using amylose resin (NEB) according to the manufacturer's recommendations.

Recombinant full-length *Drosophila melanogaster* KANSL1, KANSL3 and MOF were expressed with an N-terminal 3FLAG/HA tag using the Bac-to-Bac (Invitrogen) system in SF21 cells. Fifty millilitres of infected cells were harvested by centrifugation, washed in PBS and resuspended in high Tween20 HKMGT buffer (25 mM HEPES pH 7.6, 150 mM KCl, 12.5 mM MgCl_2_, 10% glycerol, 0.4% Tween20) with Complete protease inhibitors (Roche) added. Following three freeze thaw cycles between liquid nitrogen and a 20 °C water bath, the lysate was ultracentrifuged at 100,000*g* for 30 min at 4 °C. The cleared lysate was incubated with 80 μl pre-washed FLAG beads (Sigma) overnight. Following one wash in the above HKMGT buffer and three in a low Tween20 (0.1%) HKMGT buffer, the samples were eluted using 250 μg ml^−1^ of 3XFLAG peptide (Sigma) in low Tween20 HKMGT with protease inhibitors added. Proteins were quantified by Coomassie blue staining and snap frozen in aliquots.

### Beads experiments in *Xenopus* egg extracts

Fresh cytosolic factor (CSF)-arrested *Xenopus* EE was prepared as previously described^43^. xTPX2 depletion from *Xenopus* egg extracts was performed by two 30-min rounds of depletion using homemade anti-xTPX2 antibodies coupled with Protein A dynabeads (Life Technologies). Recombinant KANSL1, KANSL3, MOF and xMCRS1 were incubated in CSF-egg extract at 100 nM final concentration for 30 min at 20 °C with or without addition of 10 μM RanGTPQ69L. Protein A-coated Dynabeads (Invitrogen) were washed three times in PBS 0.1% Triton X100 (Sigma) and incubated with the indicated antibodies (anti-HA for KANSL1, KANSL3 and MOF; anti-MBP for xMCRS1) diluted in the same buffer for 1 h at room temperature, following the manufacturer's indications. Beads were retrieved on a magnet and washed twice in PBS 0.1% Triton X100 and twice in CSF-XB (10 mM HEPES; 100 mM KCl; 0,1 mM CaCl_2_, 2 mM MgCl_2_; 50 mM sucrose; 5 mM EGTA). Beads were then incubated in CSF-egg extract (at 1:3) for 1 h at 4 °C. Beads were recovered on a magnet, washed twice in CSF-XB and twice in BRB80 (80 mM PIPES; 1 mM MgCl_2_; 5 mM EGTA; pH 6.8) and resuspended in BRB80. They were eventually incubated in the presence of 5 μM importin-β. Two microlitres of beads was added to 30 μl of pure tubulin (20 μM tubulin purified from calf brain, BRB80, 1 mM GTP) and incubated at 37 °C for 10 min. The sample was fixed in 500 μl 1% glutaraldehyde in BRB80, and centrifuged onto a coverslip through a 10% glycerol cushion (vol/vol in BRB80) at 1,500*g* for 10 min. Coverslips were postfixed for 10 min in −20 °C methanol before processing for immunofluorescence^44^.

To evaluate microtubule efficiency around the beads, the number of beads associated with a microtubule was quantified. At least 300 beads were counted in each condition. For the western blot analysis in Fig. 2c, all IPs were performed at least three times.

### Stable isotope labelling of amino acids in cell culture

HEK293 cells stably expressing FLAG-MCRS1 were grown in SILAC DMEM R6K8 medium (Dundee cell products); control HEK293 cells were grown in SILAC DMEM R0K0 medium (Dundee cell products), both media were supplemented with 2 mM L-glutamine (Invitrogen), 10% fetal bovine serum (Dundee cell products) and 1 × penicillin and streptomycin (Invitrogen). Cells were grown for 10 days with 5% CO_2_ in a humid atmosphere on a poly-D-lysine layer (Sigma) with media changed every 48 h. Cells were synchronized over a 48-h period using first a 16-h incubation with 2 mM thymidine (Sigma), release into fresh medium for 8 h, arrest with 2 μM nocodazole (Sigma) for 16 h, followed by a 30-min release. Cells were collected by shake-off. Cell pellets were resuspended in extraction buffer (20 mM HEPES pH 7.8; 175 mM NaCl; 2,5 mM MgCl_2_; 10% glycerol; 1 mM dithiothreitol (DTT; Sigma); 1 mM phenylmethylsulphonyl fluoride (Sigma); protease inhibitor cocktail (Roche); 2 μM okadaic acid (Sigma); 1 mM orthovanadate (Sigma); 40 mM glycerophosphate (Sigma); and 50 mM NaF (Sigma)). Cells were lysed using a nitrogen bomb at 1,500 p.s.i. for 15 min.

An equal amount of protein (51.15 mg) of control and tagged protein was mixed and a FLAG pull-down was carried out using an ANTI-FLAG M2 affinity gel (Sigma).

The protein extract was incubated with the ANTI-FLAG M2 affinity gel for 2 h at 4 °C and washed four times with 50 mM Tris pH 7.4; 150 mM NaCl; 1 mM EDTA and 1% IGEPAL CA-630 (Sigma). FLAG proteins were eluted from the resin by competition with 400 μg ml^−1^ of FLAG peptide (Sigma). FLAG proteins were concentrated in a speedVac, separated by SDS–polyacrylamide gel electrophoresis and visualized using the NOVEX colloidal blue staining kit (Invitrogen). Bands were cut out and analysed by mass spectrometry at the Centre for Genomic Regulation proteomics facility.

### Microtubule regrowth and cold-stable assays

For microtubule regrowth assays, 3 μM nocodazole (Sigma) was added to the cell medium for 3 h, and washed out three times in PBS and once in medium at 37 °C. Cells were fixed at 5 min after nocodazole washout, and processed for immunofluorescence as described. Results were quantified counting the number of microtubule asters in at least 100 cells to obtain the average per cell.

For K-fibre length assays, cells were incubated on ice for 10 min in L15 medium (Sigma) supplemented with 20 mM HEPES (Sigma) at pH 7.3 and washed once with cold PBS before fixation. To measure K-fibre length, cells were treated with 2 μM STLC (Sigma) for 3 h and then subjected to the cold treatment in the presence of STLC. K-fibres of at least 40 monopolar spindles in each condition were measured using ImageJ.

### Microtubule co-pelleting experiment

Taxol microtubules were prepared incubating 20 μM of bovine tubulin at 37 °C in BRB80+1 mM GTP. After 5-min incubation, taxol was then progressively added at the following final concentrations: 0.2, 2 and 20 μM.

Three-micromolar taxol-stabilized microtubules were incubated with 250 nM of recombinant KANSL1, KANSL3 or *Drosophila* MOF, as indicated, for 30 min at 4 °C. Microtubules were then pelleted through a 30% glycerol cushion in 5 μM taxol in BRB80 at 100,000*g* in a TLA-100 rotor (Beckman-Coulter) for 24 min at 22 °C. Supernatant and pellet fractions were separated by SDS–polyacrylamide gel electrophoresis and subjected to western blot analysis.

### *In vitro* experiments with taxol-stabilized microtubules

For localization studies, microtubules stabilized with 1.5 μM taxol were incubated for 10 min at room temperature with 100 nM MBP-xMCRS1, KANSL1 or KANSL3, as indicated, in BRB80. Samples were then fixed with 1% glutaraldehyde, 5 μM taxol in BRB80 and pelleted on coverslips through a 25% glycerol, BRB80 cushion at 4,000*g* for 10 min at room temperature. Coverslips were then recovered and fixed for 10 min in −20 °C methanol and processed for immunofluorescence.

### *In vitro* experiments with polarity-marked microtubules

Polarity-marked microtubules were prepared from microtubule bright seeds. The seeds were prepared by mixing 1:1 Rhodamine–tubulin, unlabelled tubulin in BRB80, 1 mM GMPCPP (Jena Biosciences), 1 mM DTT and incubated 10 min on ice before centrifugation at 100,000*g* for 10 min at 4 °C. Polymerization was performed by incubation 25 min at 37 °C. Polymerized seeds were then diluted five times with 20 μM unlabelled tubulin, 1 mM GTP, 1 mM DTT in BRB80 and incubated at 37 °C for 20 min. The polarity-marked microtubules were then stabilized adding 10 μM taxol and used for *in vitro* visualization experiments.

### KANSL complex reconstitution *in vitro*

Two baculovirus constructs were created in which all seven members of the KANSL complex were overexpressed under alternating viral p10 and pH promoters. KANSL1, KANSL2, MOF, MCRS1/2, PHF20/MBD-R2 and WDR5/WDS were in their native state (untagged). KANSL3 was tagged with 3FLAG to enable it to function as the bait. SF21 cells co-infected with the two viruses produce high levels of all the protein members simultaneously. After incubating the extract (see ‘Recombinant proteins' for preparation) from cells infected with these viruses with FLAG beads, a complete KANSL complex was purified. This was performed individually for both human and *Drosophila* complexes.

## Additional information

**How to cite this article:** Meunier, S. *et al.* An epigenetic regulator emerges as microtubule minus-end binding and stabilizing factor in mitosis. *Nat. Commun.* 6:7889 doi: 10.1038/ncomms8889 (2015).

## Supplementary Material

Supplementary Figures Supplementary Figures 1-5

Supplementary Movie 1 An example of a HeLa cells subjected to a scrambled/control siRNA completing a normal mitosis. Live cell imaging of a typical cell undergoing mitosis following siScrambled knockdown (control). HeLa cells co-expressing GFP-tubulin and H2B-mCherry were used. Time lapse was acquired using a 63X objective, with a stack taken every 3 minutes. The movie is presented as a maximum intensity projection of ten slices. The movie is played at 20 frames per second. Time indicated is in minutes.

Supplementary Movie 2 An example of a HeLa cell subjected to KANSL1 siRNA exhibiting misaligned chromosomes. Live cell imaging of a typical mitotic defect exhibited following KANSL1 knockdown. HeLa cells co-expressing GFP-tubulin and H2B-mCherry were used. Time lapse was acquired using a 63X objective, with a stack taken every 3 minutes. The movie is presented as a maximum intensity projection of ten slices. The movie is played at 20 frames per second. Time indicated is in minutes

Supplementary Movie 3 An example of a HeLa cell subjected to KANSL3 siRNA exhibiting misaligned chromosomes. Live cell imaging of a typical mitotic defect exhibited following KANSL3 knockdown. HeLa cells co-expressing GFP-tubulin and H2B-mCherry were used. 5 Time lapse was acquired using a 63X objective, with a stack taken every 3 minutes. The movie is presented as a maximum intensity projection of ten slices. The movie is played at 20 frames per second. Time indicated is in minutes. Time indicated is in minutes.

Supplementary Movie 4 HeLa cells subjected to a scrambled/control siRNA rarely show mitotic defects. Live cell imaging of a group of cells undergoing mitosis following siScrambled knockdown (control). HeLa cells co-expressing GFP-tubulin and H2B-mCherry were used. Time lapse was acquired using a 40X objective, with a stack taken every 8 minutes. A single slice of the stack is presented. The movie is played at 10 frames per second. Time indicated is in minutes.

Supplementary Movie 5 HeLa cells subjected to KANSL1 siRNA show frequent mitotic defects. Live cell imaging showing the range of mitotic defects exhibited following KANSL1 knockdown. HeLa cells co-expressing GFP-tubulin and H2B-mCherry were used. Time lapse was acquired using a 40X objective, with a stack taken every 8 minutes. A single slice of the stack is presented. The movie is played at 10 frames per second. Time indicated is in minutes.

Supplementary Movie 6 HeLa cells subjected to KANSL3 siRNA show frequent mitotic defects. 6 Live cell imaging showing the range of mitotic defects exhibited following KANSL3 knockdown. HeLa cells co-expressing GFP-tubulin and H2B-mCherry were used. Time lapse was acquired using a 40X objective, with a stack taken every 8 minutes. A single slice of the stack is presented. The movie is played at 10 frames per second. Time indicated is in minutes.

## References

[b1] Cai Y. *et al.* Subunit composition and substrate specificity of a MOF-containing histone acetyltransferase distinct from the male-specific lethal (MSL) complex. J. Biol. Chem. 285, 4268–4272 (2010). 20018852 10.1074/jbc.C109.087981 PMC2836030

[b2] Dias J. *et al.* Structural analysis of the KANSL1/WDR5/KANSL2 complex reveals that WDR5 is required for efficient assembly and chromatin targeting of the NSL complex. Genes Dev. 28, 929–942 (2014). 24788516 10.1101/gad.240200.114 PMC4018492

[b3] Mendjan S. *et al.* Nuclear pore components are involved in the transcriptional regulation of dosage compensation in Drosophila. Mol. Cell 21, 811–823 (2006). 16543150 10.1016/j.molcel.2006.02.007

[b4] Feller C. *et al.* The MOF-containing NSL complex associates globally with housekeeping genes, but activates only a defined subset. Nucleic Acids Res. 40, 1509–1522 (2012). 22039099 10.1093/nar/gkr869 PMC3287193

[b5] Lam K. C. *et al.* The NSL complex regulates housekeeping genes in Drosophila. PLoS Genet. 8, e1002736 (2012). 22723752 10.1371/journal.pgen.1002736 PMC3375229

[b6] Raja S. J. *et al.* The nonspecific lethal complex is a transcriptional regulator in Drosophila. Mol. Cell 38, 827–841 (2010). 20620954 10.1016/j.molcel.2010.05.021

[b7] Chelmicki T. *et al.* MOF-associated complexes ensure stem cell identity and Xist repression. Elife 3, e02024 (2014). 24842875 10.7554/eLife.02024 PMC4059889

[b8] Ravens S. *et al.* MOF-associated complexes have overlapping and unique roles in regulating pluripotency in embryonic stem cells and during differentiation. Elife, e02104 (2014). 10.7554/eLife.02104 PMC4059888 24898753

[b9] Koolen D. A. & de Vries B. B. A. KANSL1-Related Intellectual Disability Syndrome eds Pagon R. A.*et al.* GeneReviews (1993).

[b10] Koolen D. A. *et al.* Mutations in the chromatin modifier gene KANSL1 cause the 17q21.31 microdeletion syndrome. Nat. Genet. 44, 639–641 (2012). 22544363 10.1038/ng.2262

[b11] Zollino M. *et al.* Mutations in KANSL1 cause the 17q21.31 microdeletion syndrome phenotype. Nat. Genet. 44, 636–638 (2012). 22544367 10.1038/ng.2257

[b12] Gilissen C. *et al.* Genome sequencing identifies major causes of severe intellectual disability. Nature 511, 344–347 (2014). 24896178 10.1038/nature13394

[b13] Gottesfeld J. M. & Forbes D. J. Mitotic repression of the transcriptional machinery. Trends Biochem. Sci. 22, 197–202 (1997). 9204705 10.1016/s0968-0004(97)01045-1

[b14] Kruhlak M. J. *et al.* Regulation of global acetylation in mitosis through loss of histone acetyltransferases and deacetylases from chromatin. J. Biol. Chem. 276, 38307–38319 (2001). 11479283 10.1074/jbc.M100290200

[b15] Karsenti E. & Vernos I. The mitotic spindle: a self-made machine. Science 294, 543–547 (2001). 11641489 10.1126/science.1063488

[b16] Kalab P., Weis K. & Heald R. Visualization of a Ran-GTP gradient in interphase and mitotic *Xenopus* egg extracts. Science 295, 2452–2456 (2002). 11923538 10.1126/science.1068798

[b17] Meunier S. & Vernos I. Microtubule assembly during mitosis - from distinct origins to distinct functions? J. Cell Sci. 125, 2805–2814 (2012). 22736044 10.1242/jcs.092429

[b18] Yokoyama H & Gruss O. J. New mitotic regulators released from chromatin. Front. Oncol. 3, 308 (2013). 24380075 10.3389/fonc.2013.00308 PMC3864359

[b19] Yokoyama H., Rybina S., Santarella-Mellwig R., Mattaj I. W. & Karsenti E. ISWI is a RanGTP-dependent MAP required for chromosome segregation. J. Cell Biol. 187, 813–829 (2009). 20008562 10.1083/jcb.200906020 PMC2806316

[b20] Neumann B. *et al.* Phenotypic profiling of the human genome by time-lapse microscopy reveals cell division genes. Nature 464, 721–727 (2010). 20360735 10.1038/nature08869 PMC3108885

[b21] Masui Y. & Clarke H. J. Oocyte maturation. Int. Rev. Cytol. 57, 185–282 (1979). 385540 10.1016/s0074-7696(08)61464-3

[b22] Wuhr M. *et al.* Deep proteomics of the *Xenopus* laevis egg using an mRNA-derived reference database. Curr. Biol. 24, 1467–1475 (2014). 24954049 10.1016/j.cub.2014.05.044 PMC4090281

[b23] Scrofani J., Sardon T., Meunier S. & Vernos I. Microtubule nucleation in mitosis by a RanGTP-dependent protein complex. Curr. Biol. 25, 131–140 (2015). 25532896 10.1016/j.cub.2014.11.025

[b24] Schatz C. A. *et al.* Importin alpha-regulated nucleation of microtubules by TPX2. EMBO J. 22, 2060–2070 (2003). 12727873 10.1093/emboj/cdg195 PMC156067

[b25] Meunier S. & Vernos I. K-fibre minus ends are stabilized by a RanGTP-dependent mechanism essential for functional spindle assembly. Nat. Cell Biol. 13, 1406–1414 (2011). 22081094 10.1038/ncb2372

[b26] Tulu U. S., Fagerstrom C., Ferenz N. P. & Wadsworth P. Molecular requirements for kinetochore-associated microtubule formation in mammalian cells. Curr. Biol. 16, 536–541 (2006). 16527751 10.1016/j.cub.2006.01.060 PMC1500889

[b27] Rieder C. L. & Borisy G. G. The attachment of kinetochores to the pro-metaphase spindle in ptk1 cells - recovery from low-temperature treatment. Chromosoma 82, 693–716 (1981). 7261715 10.1007/BF00285776

[b28] Maresca T. J. & Salmon E. D. Intrakinetochore stretch is associated with changes in kinetochore phosphorylation and spindle assembly checkpoint activity. J. Cell Biol. 184, 373–381 (2009). 19193623 10.1083/jcb.200808130 PMC2646557

[b29] Galjart N. Plus-end-tracking proteins and their interactions at microtubule ends. Curr. Biol. 20, R528–R537 (2010). 20620909 10.1016/j.cub.2010.05.022

[b30] Tanaka N., Meng W., Nagae S. & Takeichi M. Nezha/CAMSAP3 and CAMSAP2 cooperate in epithelial-specific organization of noncentrosomal microtubules. Proc. Natl Acad. Sci. USA 109, 20029–20034 (2012). 23169647 10.1073/pnas.1218017109 PMC3523837

[b31] Richardson C. E. *et al.* PTRN-1, a microtubule minus end-binding CAMSAP homolog, promotes microtubule function in Caenorhabditis elegans neurons. Elife 3, e01498 (2014). 24569477 10.7554/eLife.01498 PMC3932522

[b32] Meng W., Mushika Y., Ichii T. & Takeichi M. Anchorage of microtubule minus ends to adherens junctions regulates epithelial cell-cell contacts. Cell 135, 948–959 (2008). 19041755 10.1016/j.cell.2008.09.040

[b33] Marcette J. D., Chen J. J. & Nonet M. L. The Caenorhabditis elegans microtubule minus-end binding homolog PTRN-1 stabilizes synapses and neurites. Elife 3, e01637 (2014). 24569480 10.7554/eLife.01637 PMC3930908

[b34] Goshima G. *et al.* Genes required for mitotic spindle assembly in Drosophila S2 cells. Science 316, 417–421 (2007). 17412918 10.1126/science.1141314 PMC2837481

[b35] Nagae S., Meng W. & Takeichi M. Non-centrosomal microtubules regulate F-actin organization through the suppression of GEF-H1 activity. Genes Cells 18, 387–396 (2013). 23432781 10.1111/gtc.12044

[b36] Goodwin S. S. & Vale R. D. Patronin regulates the microtubule network by protecting microtubule minus ends. Cell 143, 263–274 (2010). 20946984 10.1016/j.cell.2010.09.022 PMC3008421

[b37] Hyman A. A. Preparation of marked microtubules for the assay of the polarity of microtubule-based motors by fluorescence. J. Cell Sci. Suppl. 14, 125–127 (1991). 1832165 10.1242/jcs.1991.supplement_14.25

[b38] Hendershott M. C. & Vale R. D. Regulation of microtubule minus-end dynamics by CAMSAPs and Patronin. Proc. Natl Acad. Sci. USA 111, 5860–5865 (2014). 24706919 10.1073/pnas.1404133111 PMC4000804

[b39] Wang H., Brust-Mascher I., Civelekoglu-Scholey G. & Scholey J. M. Patronin mediates a switch from kinesin-13-dependent poleward flux to anaphase B spindle elongation. J. Cell Biol. 203, 35–46 (2013). 24100293 10.1083/jcb.201306001 PMC3798244

[b40] Jiang K. *et al.* Microtubule minus-end stabilization by polymerization-driven CAMSAP deposition. Dev. Cell 28, 295–309 (2014). 24486153 10.1016/j.devcel.2014.01.001

[b41] Piel M., Meyer P., Khodjakov A., Rieder C. L. & Bornens M. The respective contributions of the mother and daughter centrioles to centrosome activity and behavior in vertebrate cells. J. Cell Biol. 149, 317–330 (2000). 10769025 10.1083/jcb.149.2.317 PMC2175166

[b42] Dunsch A. K., Linnane E., Barr F. A. & Gruneberg U. The astrin-kinastrin/SKAP complex localizes to microtubule plus ends and facilitates chromosome alignment. J. Cell Biol. 192, 959–968 (2011). 21402792 10.1083/jcb.201008023 PMC3063133

[b43] Sardon T., Peset I., Petrova B. & Vernos I. Dissecting the role of Aurora A during spindle assembly. EMBO J. 27, 2567–2579 (2008). 18756265 10.1038/emboj.2008.173 PMC2567403

[b44] Peset I. *et al.* Function and regulation of Maskin, a TACC family protein, in microtubule growth during mitosis. J. Cell Biol. 170, 1057–1066 (2005). 16172207 10.1083/jcb.200504037 PMC2171525

