# Supplementary material for: An epigenetic regulator emerges as microtubule minus-end binding and stabilizing factor in mitosis
Source: Nat Commun. 2015 Aug 5;6:7889. doi: 10.1038/ncomms8889 (PMC4918316; doi:10.1038/ncomms8889)
Supplement: Supplementary Figures — 1-5 [file ncomms8889-s1.pdf]

Supplementary Figure 1

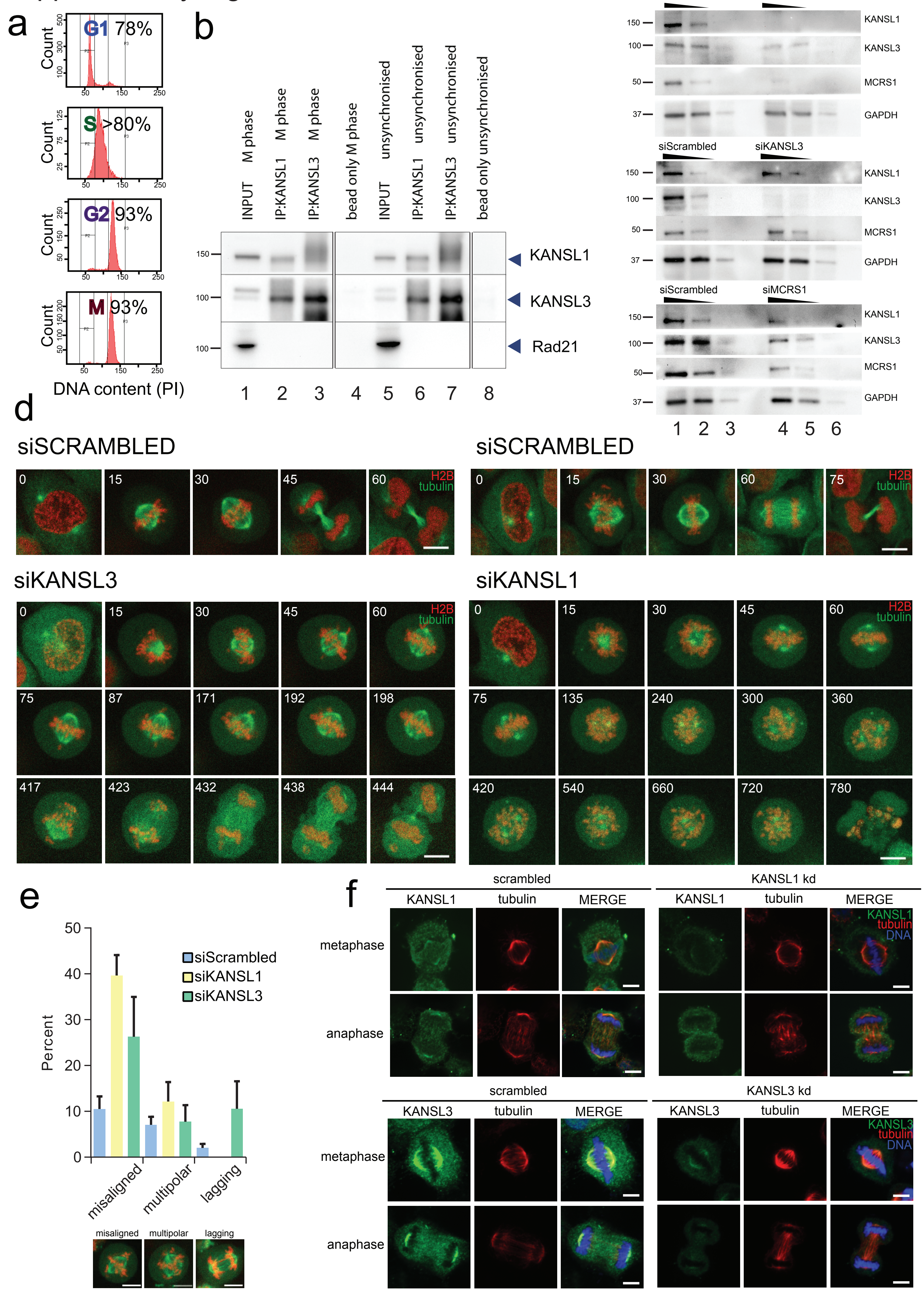

Supplementary Figure 1. KANSL1 and KANSL3 localize to spindle poles in mitosis and their depletion leads to mitotic defects.

a. Cell synchronization was assessed by FACS analysis following propidium iodide staining. Propidium iodide was excited with the 561nm laser and collected through the 610/20 BP filter in detector A. 10,000-15,000 cells were measured per condition.

b. KANSL1 and KANSL3 interact in mitosis. Western blots of the immunoprecipitations of KANSL1 and KANSL3 endogenous proteins from nocodazole-synchronized and unsynchronized HeLa cells. KANSL1 and KANSL3 were able to pull down each other with equal efficiency in both metaphase and interphase cells. The specific KANSL1, KANSL3 and Rad21 bands are indicated. Uncropped Western blots are presented in Supplementary Figure 5b.

c. Western blot analysis showing the efficiency of KANSL3, KANSL1 and MCRS1 siRNA silencing at the protein level and the impact of knockdown on the stability of other complex members. A dilution series at 100%, 50% and 10% (from left to right) was loaded for each sample. Knockdown efficiency is estimated at approximately 82% for KANSL1, 79% for KANSL3 and 70% for MCRS1 (mean of two experiments). Uncropped Western blots are presented in Supplementary Figure 5c.

d. Expanded time points of the still images of the movies shown in Figure 1b. Time in minutes is indicated in the upper left corners. Movies are in supplemental material. Scale bars, 10μm.

e. Frequency of the three main mitotic defects exhibited by cells following KANSL1 and KANSL3 knockdown. An example of each phenotype is illustrated below (Scale bars, 10μm). Percentages indicate cells exhibiting the phenotype as a proportion of all cells seen in the movie. Phenotypes are not mutually exclusive, so a cell that is counted as having exhibited misaligned chromosomes can also be counted as exhibiting multipolar spindles. Error bars, SEM.

f. Immunofluorescence analysis on control and silenced HeLa cells to show the specificity of the anti KANSL1 and KANSL3 antibodies. Tubulin is in red and DNA is in blue. Scale bars, 5μm.

Supplementary Figure 2

a

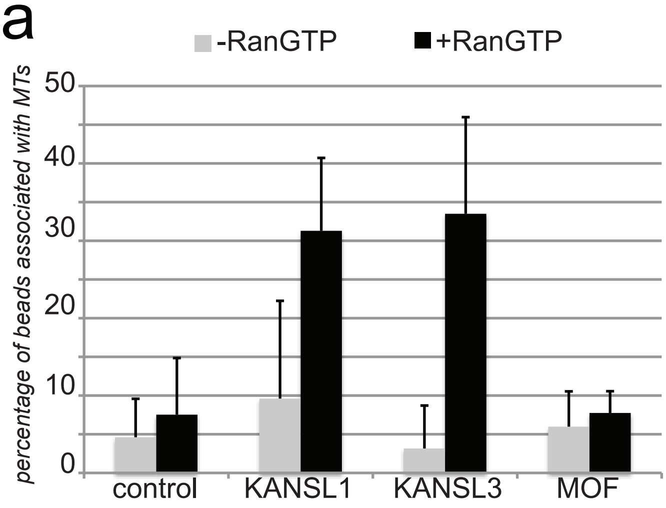

b

protein	*Ratio H/L*	*ranking*
MCRS1	68	1
KANSL3	30	2
KANSL1	25	3
KANSL2	25	4
PHF20	23	5
MOF	15	6
HCFC1	11	10
WDR5	3,2	36

c

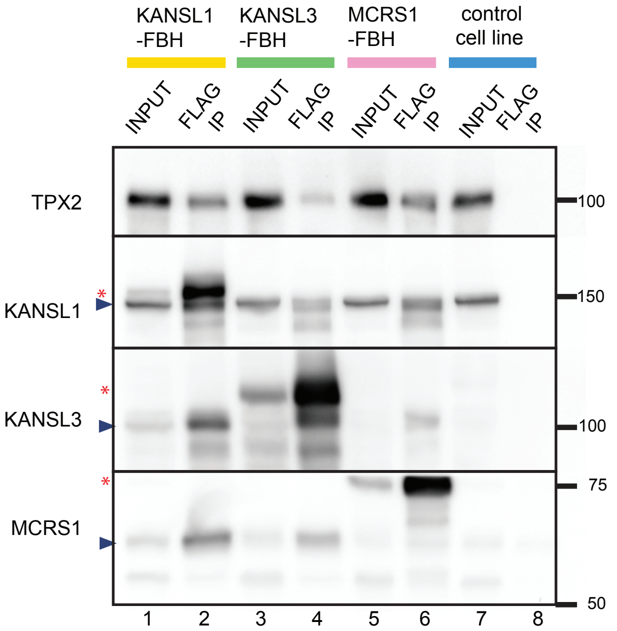

d

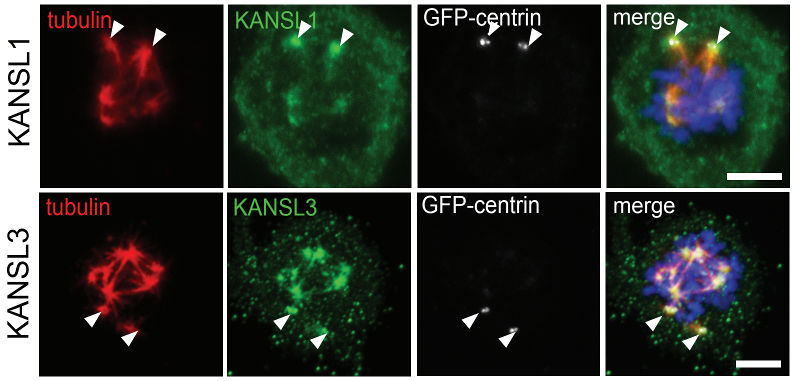

e

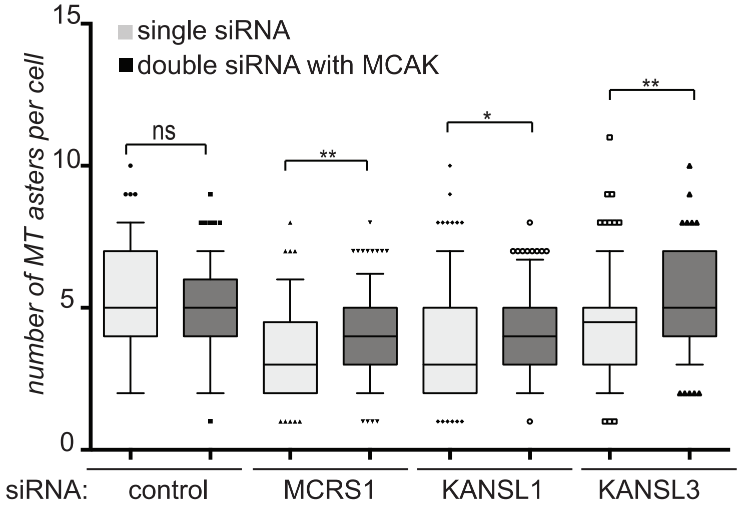

f

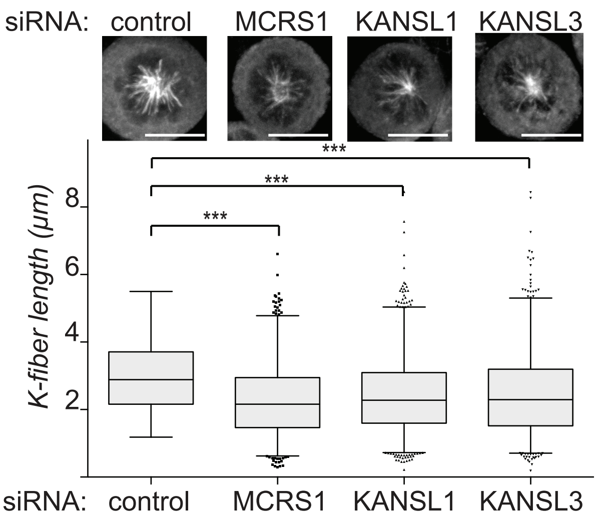

Supplementary Figure 2. KANSL1, KANSL3 and MCRS1 interact in mitotic cells and function together in the Ran pathway of microtubule assembly.

a. MOF does not induce MT assembly in pure tubulin. Recombinant MOF, KANSL1 or KANSL3 were incubated in egg extract +/- RanGTP and retrieved on magnetic beads. The beads were then washed and incubated in 20µM pure tubulin and spun down on coverslips for immunofluorescence analysis. The graph shows the percentage of beads associated with MTs after 10min 37˚C incubation. Bars: SD.

b. Table showing the top list of mitotic interacting partners of MCRS1 obtained by SILAC and mass spectrometry. The Ratio heavy/light (H/L) for each protein is indicated. The ranking corresponds to the scores obtained in the whole experiment for each protein.

c. Tagged NSL proteins are able to pull down hTPX2 in mitotic HEK293 extracts. A FLAG pulldown was performed on 3FLAG, biotin acceptor site, 6His (FBH)-tagged KANSL1, KANSL3 and MCRS1 synchronized cell lines. A HEK293 cell line not expressing a FLAG-tagged protein serves as the control. The FBH tag results in the addition of approximately 13kD to the protein, resulting in a mobility shift between endogenous and tagged proteins. Endogenous proteins are marked with an arrow and tagged proteins with an asterisk. Molecular weight markers are indicated on the right. Uncropped Western blots are presented in Supplementary Figure 5d.

d. KANSL1 and KANSL3 localize both to chromosomal and centrosomal asters in MT regrowth assays. Nocodazole washout was performed in GFP-centrin HeLa cells to distinguish centrosomes clearly (marked with arrowheads). Pictures are maximum intensity projections. Scale bar, 5µm.

e. MT regrowth experiment in cells co-silenced for MCAK and NSL proteins. Number of MT asters 5 minutes after nocodazole washout in more than 100 cells for each condition; n=3; one representative experiment is shown. Box and whisker plot: boxes show the upper and lower quartile, whiskers extend from the 10th to the 90th percentile and dots correspond to outliers. Light grey boxes are single siRNA conditions; dark grey boxes are double siRNA conditions (with MCAK siRNA). MCAK silencing partially rescued MCRS1, KANSL1 and KANSL3 phenotypes on the number of asters in MT regrowth: for MCRS1 silencing, 3,34

asters on average and 4,15 upon co-silencing of MCAK ; for KANSL1 silencing, 3,82 asters on average and 4,35 upon co-silencing of MCAK and for KANSL3 silencing, 4,49 asters on average and upon co-silencing of MCAK. * p<0,05; ** p<0,005 ; ns= non significant (unpaired t-test).

f. K-fiber length in control and silenced cells. Top: representative images of STLC treated control and silenced HeLa cells, after a 10min cold-induced MT depolymerization, fixed and stained with anti-tubulin antibodies. Scale bar: 5μM. Box and whisker plot: boxes show the upper and lower quartile, whiskers extend from the 10th to the 90th percentile and dots correspond to outliers. K-fibers are shorter in MCRS1- (average 2,28μm), KANSL1- (2,42μm), and KANSL3-silenced cells (2,46μm) than in controls (average 2,99μm). *** p=0,0001 (unpaired t-test). More than 791 K-fibers were measured in each condition, n=4.

Supplementary Figure 3

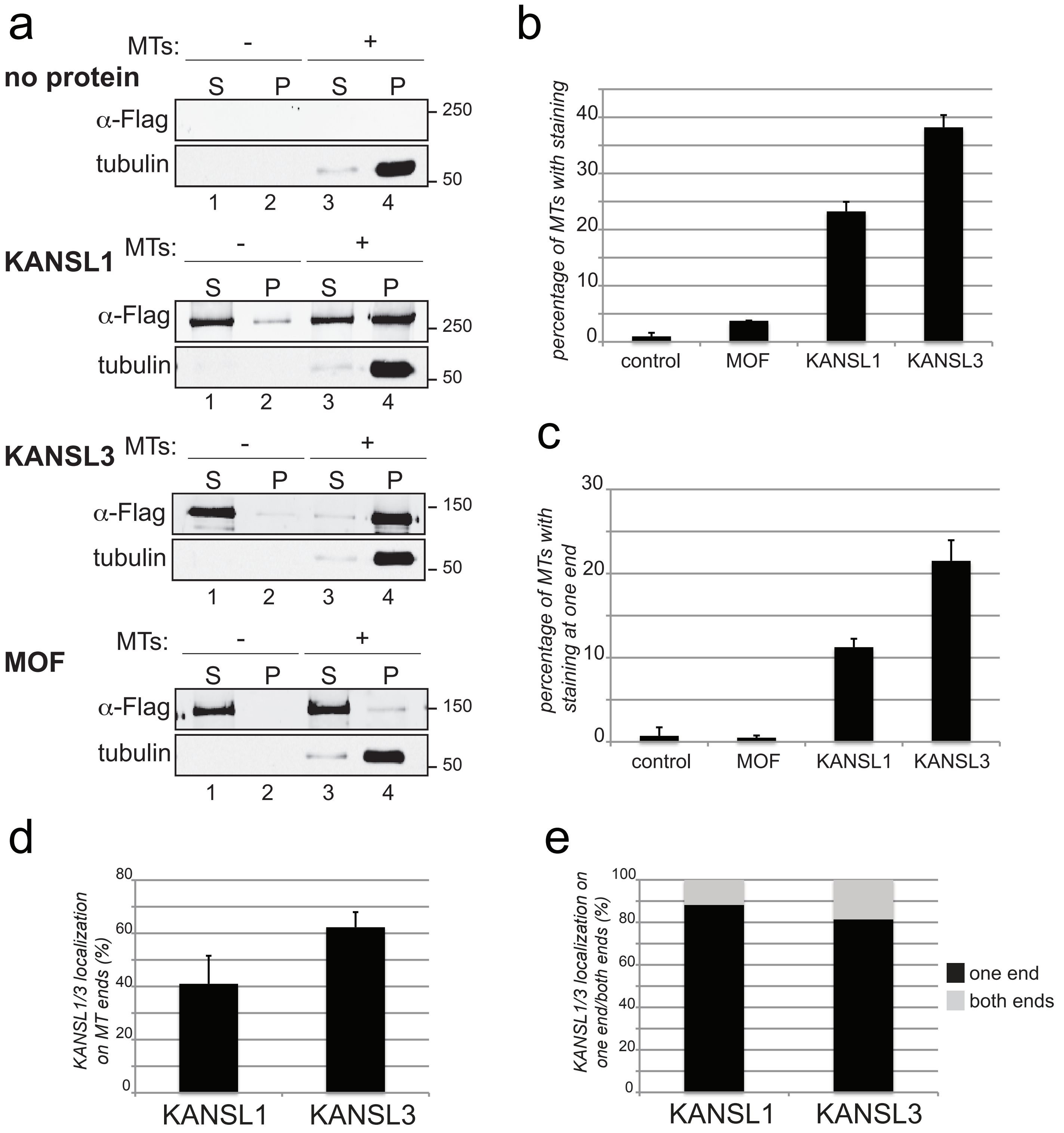

Supplementary Figure 3. Characterization of KANSL1 and KANSL3 MT binding properties *in vitro.*

a. MT co-pelleting assay. Recombinant KANSL1, KANSL3 or MOF, as indicated, were incubated with (+) or without (-) taxol-stabilized MTs. MTs were spun-down through a 50% glycerol cushion. MT pellets (P) were collected as well as supernatants (S), and loaded on SDS-PAGE. Indicated proteins (KANSL1, KANSL3 and MOF) and tubulin were detected by Western blot. KANSL1 and KANSL3 are found in the pellet only when pre-incubated with MTs showing that both proteins associate with MTs *in vitro*. Instead, MOF does not co-pellet with MTs *in vitro*.

b. Quantification from immunofluorescence images of the percentage of taxol-stabilized MTs associated with MOF, KANSL1 or KANSL3, as indicated. n=3, more than 200 MTs were counted for each condition in each experiment. Bar=sd.

c. Quantification from immunofluorescence images of the percentage of taxol-stabilized MTs having a MOF, KANSL1 or KANSL3 signal at their ends. n=3, more than 200 MTs were counted for each condition in each experiment. Bar=sd.

d. Quantification of KANSL1 and KANSL3 MT lattice versus end binding. The number of positive signals for the corresponding proteins associated to the MT lattice or the ends were recorded. Results are in percentage. KANSL1 and KANSL3 preferentially bind to MT ends. n=3, more than 200 MTs were counted for each condition in each experiment. Bar=sd.

e. Quantification of MT having a positive signal for KANSL1 or KANSL3 at one or both ends. KANSL1 and KANSL3 bind preferentially to only one MT end. n=3, more than 200 MTs were counted for each condition in each experiment.

Supplementary Figure 4

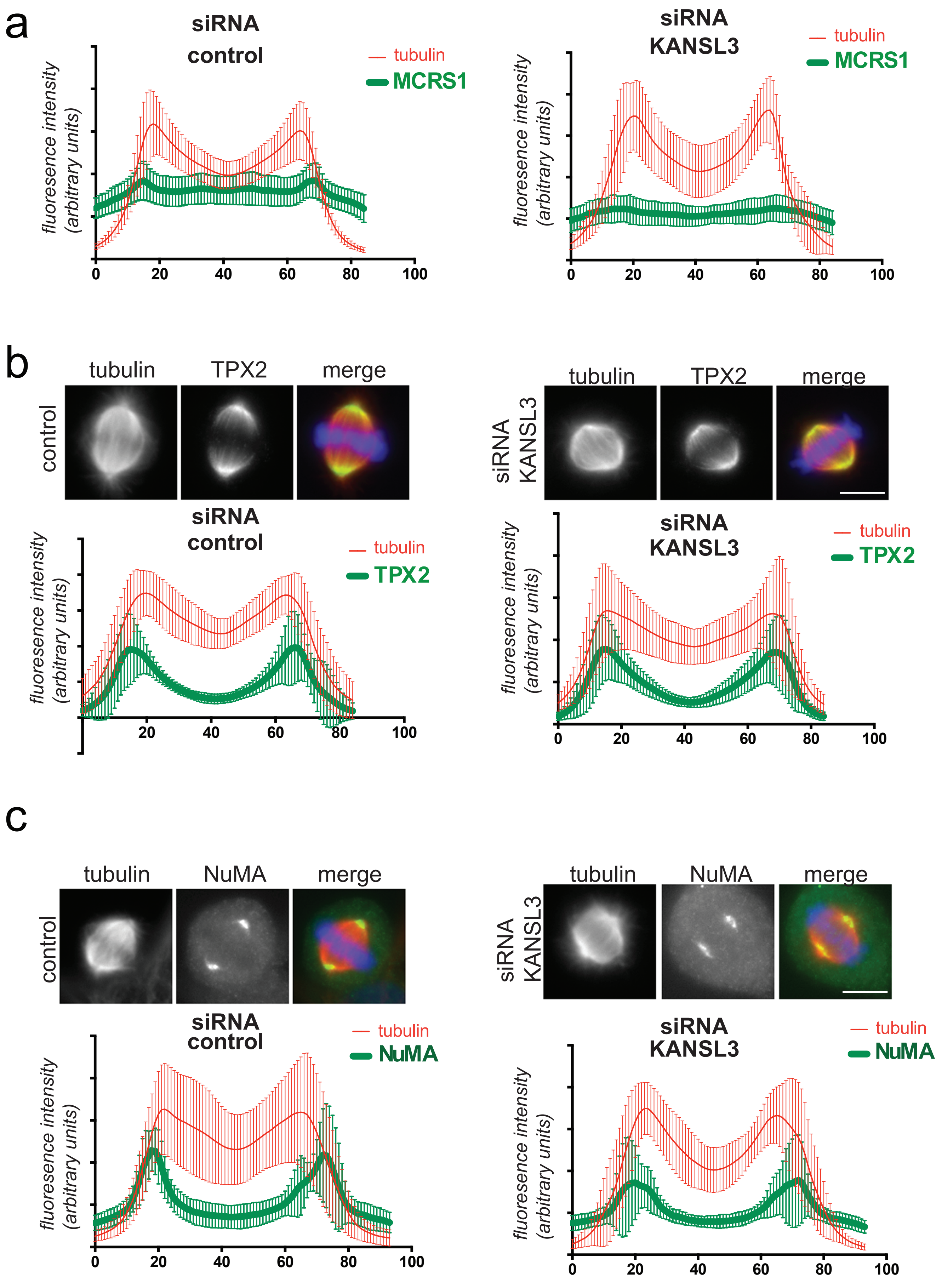

Supplementary Figure 4. Line scan analysis of MCRS1, TPX2 and NuMA localization along the spindle axis in control and KANSL3-silenced cells.

a. Quantification corresponding to the experiment shown in figure 4d for MCRS1 and tubulin. 17 spindles were quantified in each condition. Bars = sd. The two peaks corresponding to MCRS1 accumulation at spindle poles disappear in cells silenced for KANSL3.

b. Top. Representative immunofluorescence images showing tubulin (red) and TPX2 (green) in mitotic control (left) and KANSL3 silenced (right) Hela cells. Scale bar: 5µm.

Bottom. Quantification of the anti-TPX2 staining intensity in mitotic control (left) and KANSL3 silenced (right) Hela cells. The fluorescence intensity of tubulin (red) and TPX2 (green) was quantified along a straight line between the two spindle poles. 18 spindles were quantified in each condition. Bars = sd. TPX2 localization is unaffected by KANSL3 silencing.

c. Top. Representative immunofluorescence images showing tubulin (red) and NuMA (green) in mitotic control (left) or KANSL3 silenced (right) Hela cells.

Bottom. Quantification of the anti-NuMA staining intensity in mitotic control (left) and KANSL3 silenced (right) Hela cells. The fluorescence intensity of tubulin (red) and NuMA (green) was quantified along a straight line between the two spindle poles. 14 spindles were quantified in each condition. Bars = sd.

Supplementary Figure 5

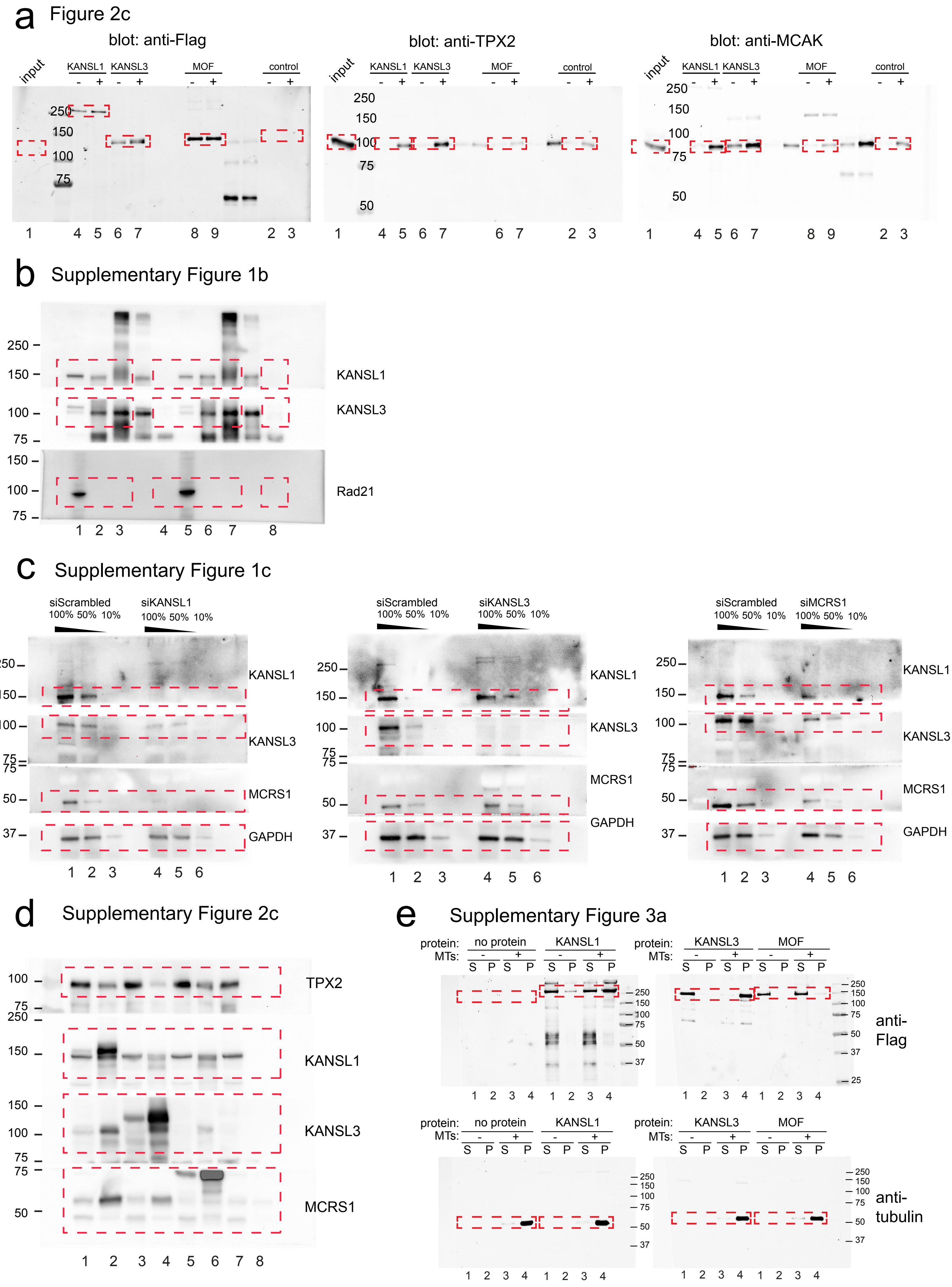

Supplementary Figure 5. Uncropped Western blot panels.

a. Uncropped Western blots from Figure 2C. Red boxes show cropped regions.

b. Uncropped Western blots from Supplementary Figure 1b. Red boxes show cropped regions.

c. Uncropped Western blots from Supplementary Figure 1c. Red boxes show cropped regions.

d. Uncropped Western blots from Supplementary Figure 2c. Red boxes show cropped regions.

e. Uncropped Western blots from Supplementary Figure 3a. Red boxes show cropped regions.
